# Solvent-free synthesis and low-temperature crystal structure of pheno­quinone (the 1:2 *p*-benzo­quinone–phenol complex)

**DOI:** 10.1107/S2056989026005414

**Published:** 2026-06-02

**Authors:** A. Timothy Royappa, Jake A. Tan, Dean H. Johnston

**Affiliations:** a Department of Chemistry University of West Florida, 11000 University Parkway Pensacola FL 32514 USA; b Department of Chemistry, Otterbein University, 1 South Grove Street, Westerville, OH 43081, USA; University of Missouri-Columbia, USA

**Keywords:** crystal structure, cocrystal, solvent-free synthesis

## Abstract

A novel solvent-free synthesis of pheno­quinone (the common name for the hydrogen-bonded 1:2 *p*-benzo­quinone–phenol complex, C_6_H_4_O_2_·2C_6_H_6_O) is reported. This ‘green’ method produces X-ray quality cocrystals of pheno­quinone as red needles within minutes. A newly redetermined and greatly improved low-temperature (100 K) crystal structure is reported. A time-dependent density functional theory examination of the charge-transfer excitation in pheno­quinone accounts for the red color of this hydrogen-bonded complex.

## Abstract 2

The solvent-free synthesis of X-ray quality crystals of pheno­quinone (1:2 *p*-benzo­phenone:phenol cocrystal) is reported, along with its crystal structure re-determined at low temperature. A detailed time-dependent density functional examination of the charge-transfer excitation of pheno­quinone accounting for its color is also provided.

## 1. Chemical context

One definition of a cocrystal is ‘a crystal that is built up out of two or more organic compounds that are, in their pure forms, solid at ambient conditions’ (Vishweshwar *et al.*, 2006 ▸). Such cocrystals are gathering increased research attention in the pharmaceutical sector because cocrystals of active pharmaceutical ingredients (APIs) may have markedly improved pharmacological and pharmacokinetic properties compared to polymorphs of the pure APIs (Yadav *et al.*, 2009 ▸; Chettri *et al.*, 2024 ▸). In addition, cocrystals are also known to improve the physicochemical properties of agrochemicals, pigments, and solid explosives (Karimi-Jafari *et al.*, 2018 ▸). Nevertheless, it is still difficult to predict *ab initio* whether two compounds will cocrystallize; therefore, it is essential to have in hand many high-quality crystal structures of cocrystals for theory development and for training of machine-learning models. As a side note, pheno­quinone is responsible for the faint pink color of impure phenol due to air oxidation (to *p*-benzo­quinone).

Early structural studies of a cocrystal were the X-ray crystal structure investigations (Wallwork & Harding, 1953 ▸; Harding & Wallwork, 1953 ▸) of pheno­quinone, which has been known for more than a century (Pratt & Gibbs, 1913 ▸). The UV-visible spectrum and kinetics of formation of pheno­quinone were reported a little later, but no structural details were elucidated (Zuev, 1956 ▸; Tronov & Sokolovich, 1956 ▸). This cocrystal was studied in much greater detail by Sakurai (1968 ▸), and the resulting structure was deposited in the Cambridge Structural Database (CSD). The room-temperature structure of pheno­quinone obtained by Sakurai was of rather low quality, however. Additionally, the crystallization conditions given by Sakurai were vague, consisting of the single, uninformative sentence, ‘Single crystals of pheno­quinone were obtained from a mixture of acetone solutions of 1:2 mol ratio of *p*-benzo­quinone and phenol.’ This method did not yield crystals in our hands. However, we found a remarkably simple, solvent-free method that resulted in X-ray quality crystals of pheno­quinone in a few minutes. Thus, the ‘green’ synthesis and subsequent structural characterization presented here is also of relevance to the modern method of hot-melt extrusion used to produce pharmaceutical cocrystals (Narala *et al.*, 2021 ▸) and to other green cocrystal synthesis methods, including mechanochemical synthesis (Braga *et al.*, 2013 ▸; Duarte *et al.*, 2016 ▸; Qi *et al.*, 2024 ▸).
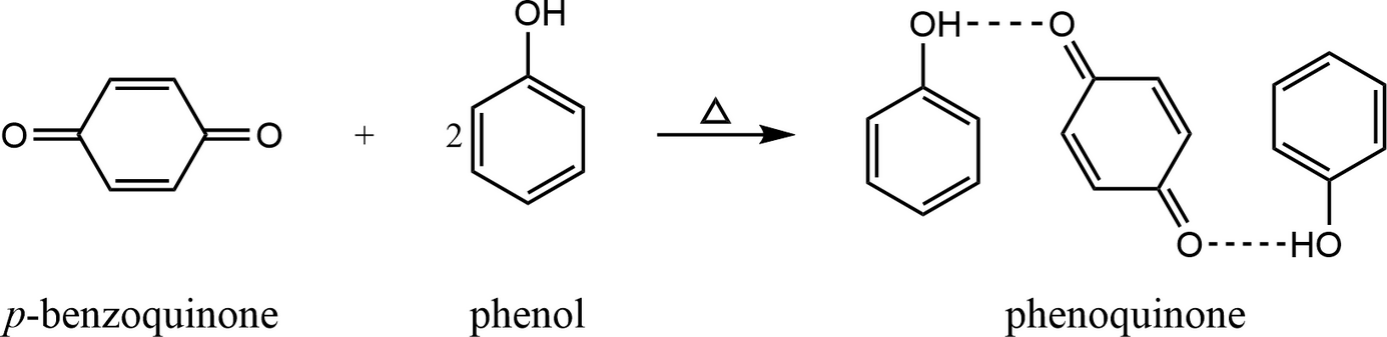


The structure described in this paper was obtained at 100 K, with multi-scan absorption correction applied and employing anisotropic displacement parameters (ADPs) for non-hydrogen atoms, resulting in much improved refinement quality compared to Sakurai’s structure (*R*_1_ = 0.0483 *vs R*_1_ = 0.1050). The lower data acquisition temperature also allowed for higher precision in geometric parameters such as bond lengths and bond angles (see the *Structural commentary* section below). The difference map showed no significant residual electron density and the ADPs were small, denoting an absence of disorder in the structure.

## 2. Structural commentary

**Figure 1:**
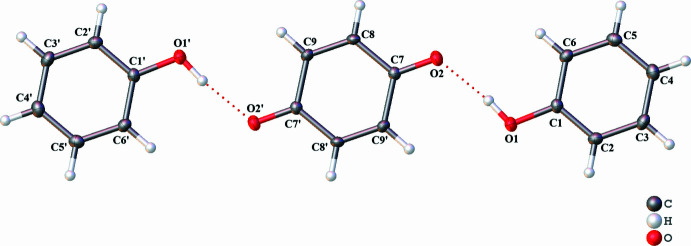
Hydrogen-bonded structure of pheno­quinone, showing 1:2 *p*-benzo­quinone:phenol stoichiometry.

**Figure 2:**
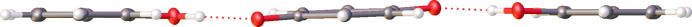
Side view of pheno­quinone showing the planar structure of the complex.

The general features of this crystal structure have been discussed in detail elsewhere (Wallwork & Harding, 1953 ▸; Harding & Wallwork, 1953 ▸; Sakurai, 1968 ▸). Briefly, each pheno­quinone unit consists of a *p*-benzo­quinone mol­ecule on an inversion center, hydrogen bonded to two centrosymmetrically arranged flanking phenols, as shown in Fig. 1 ▸. The asymmetric unit is one-half of the pheno­quinone. In this complex, the O2—H1 distance is 1.84 (2) Å [O1⋯O2 distance 2.732 (2) Å) and the O1—H1⋯O2 angle is nearly straight at 172.3 (19)°. This is an indication of much stronger hydrogen bonding than Sakurai had found (O2—H1 distance 2.05 Å, O1—H1⋯O2 angle 158°). Although the phenolic hydrogen H1 was placed in a riding position, an unmistakable electron density peak is observed ∼0.9 Å from phenolic oxygen O1, confirming that this hydrogen atom is still clearly covalently bonded to the phenolic oxygen. The three mol­ecules in each pheno­quinone complex are coplanar, as can be seen in the side view of pheno­quinone shown in Fig. 2 ▸; the r.m.s. deviation of all atoms from their mean plane is only 0.044 Å.

**Figure 3:**
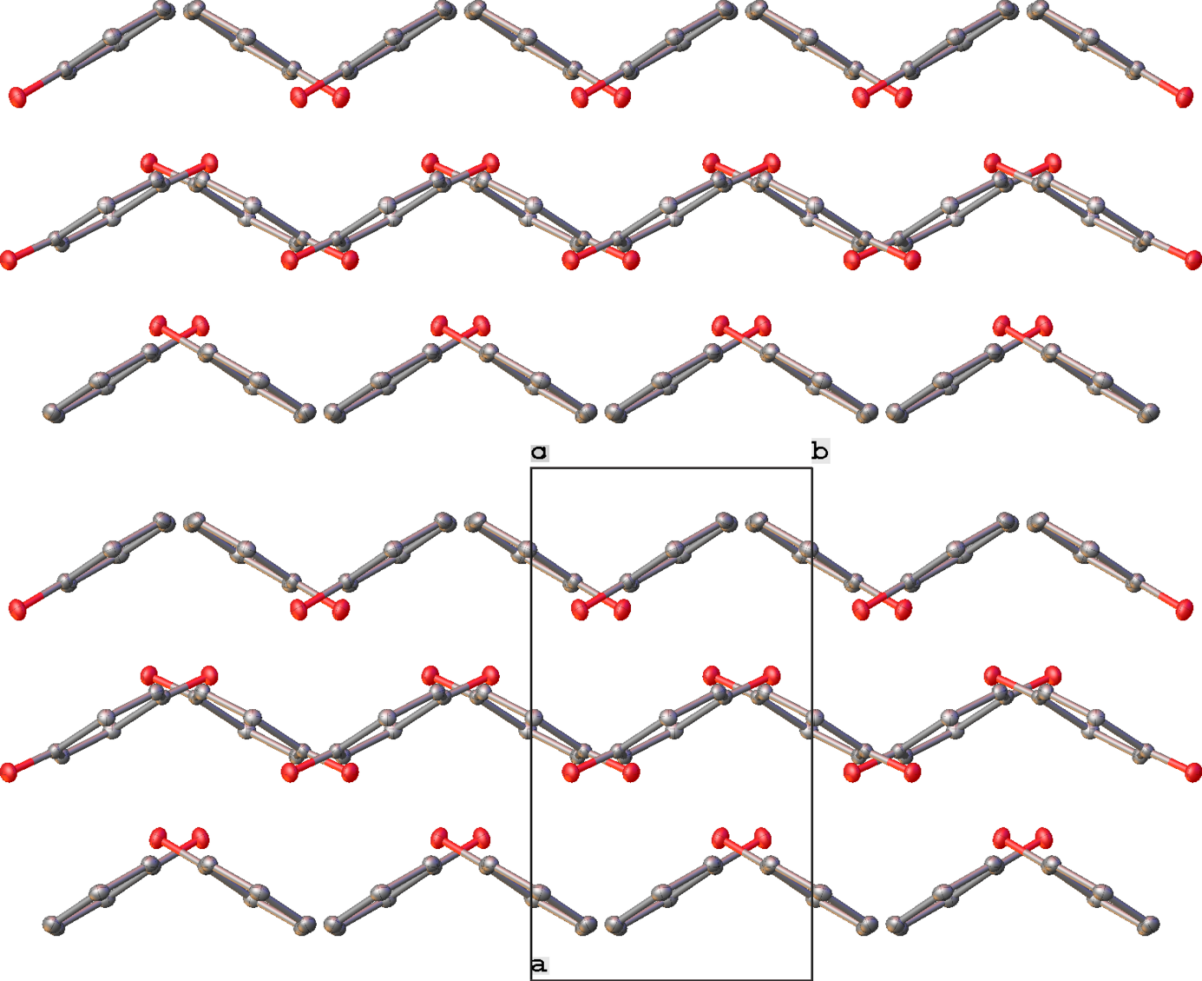
View along [001], showing the 62.5° angle between pheno­quinone planes in adjacent sheets in the solid state (H atoms omitted for clarity).

The pheno­quinone units are stacked 3.1 Å apart in columns with their planes parallel to (301), with parallel columns forming infinite sheets. These sheets alternate with sheets of identically packed pheno­quinones to generate the three-dimensional crystal structure of the solid. The angle between pheno­quinone mean planes in adjacent sheets is 62.5°, as can be seen in Fig. 3 ▸, in which the structure is viewed along [001].

## 3. Supra­molecular features

Phenol itself is colorless and *p*-benzo­quinone is yellow, whereas the cocrystals of pheno­quinone are dark red. Thus, the question immediately arises whether this red color arises from the strong hydrogen-bonding inter­actions described above or from other, weaker, crystal packing inter­actions (*e.g*., π–π inter­actions among the planar rings). However, no evidence for π–π stacking of aromatic rings was detected in this structure by the *OLEX2* software (Dolomanov *et al.*, 2009 ▸); thus, column–column and sheet–sheet packing forces are merely van der Waals inter­actions, unlikely to perturb the electronic energy levels significantly enough to cause such a drastic color change upon formation of the hydrogen-bonded complex. Moreover, as described in the *Synthesis and crystallization* section below, the melt that forms upon heating the two solids together is dark red. As weak van der Waals inter­actions are unlikely to persist in a melt at 343–344 K (Fukushima & Sakurada, 1976 ▸), the red color of solid or liquid pheno­quinone was not ascribed to such inter­actions causing changes in electronic energy levels, but to hydrogen-bonding-induced shifts in these levels. This is unusual, because hydrogen-bonding inter­actions generally manifest themselves as a red-shift in vibrational frequencies, not in altered electronic states. Indeed, the *p*-benzo­quinone C=O stretch shifted from 1671 cm^−1^ to 1638 cm^−1^ and the phenol O—H vibration from 3612 cm^−1^ to 3265 cm^−1^ upon hydrogen bonding (Fukushima & Sakurada, 1976 ▸). In each case, the free stretch in solution was compared to the hydrogen-bonded stretch in the solid state.

**Figure 4:**
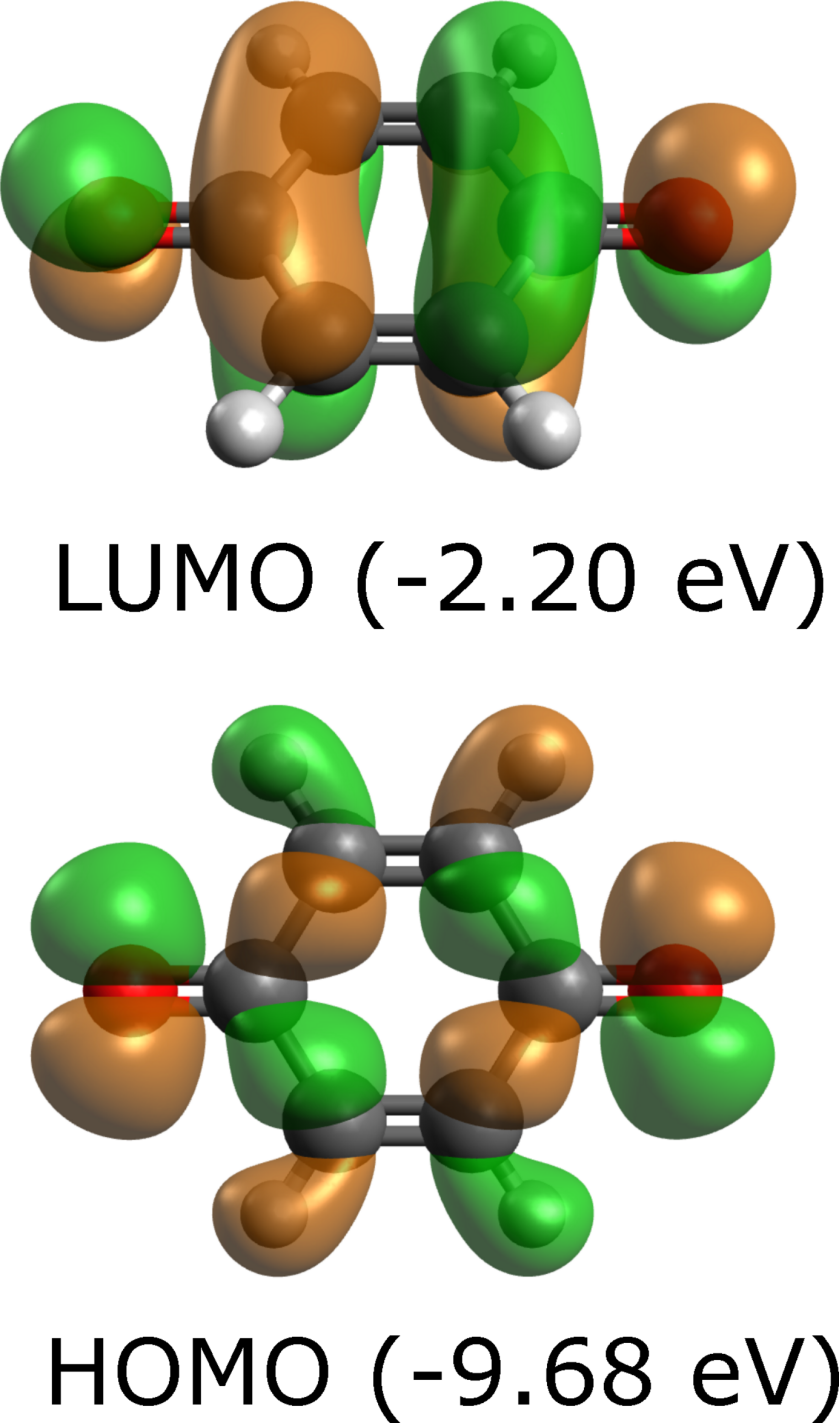
HOMO and LUMO mol­ecular orbitals of *p*-benzo­quinone.

**Figure 5:**
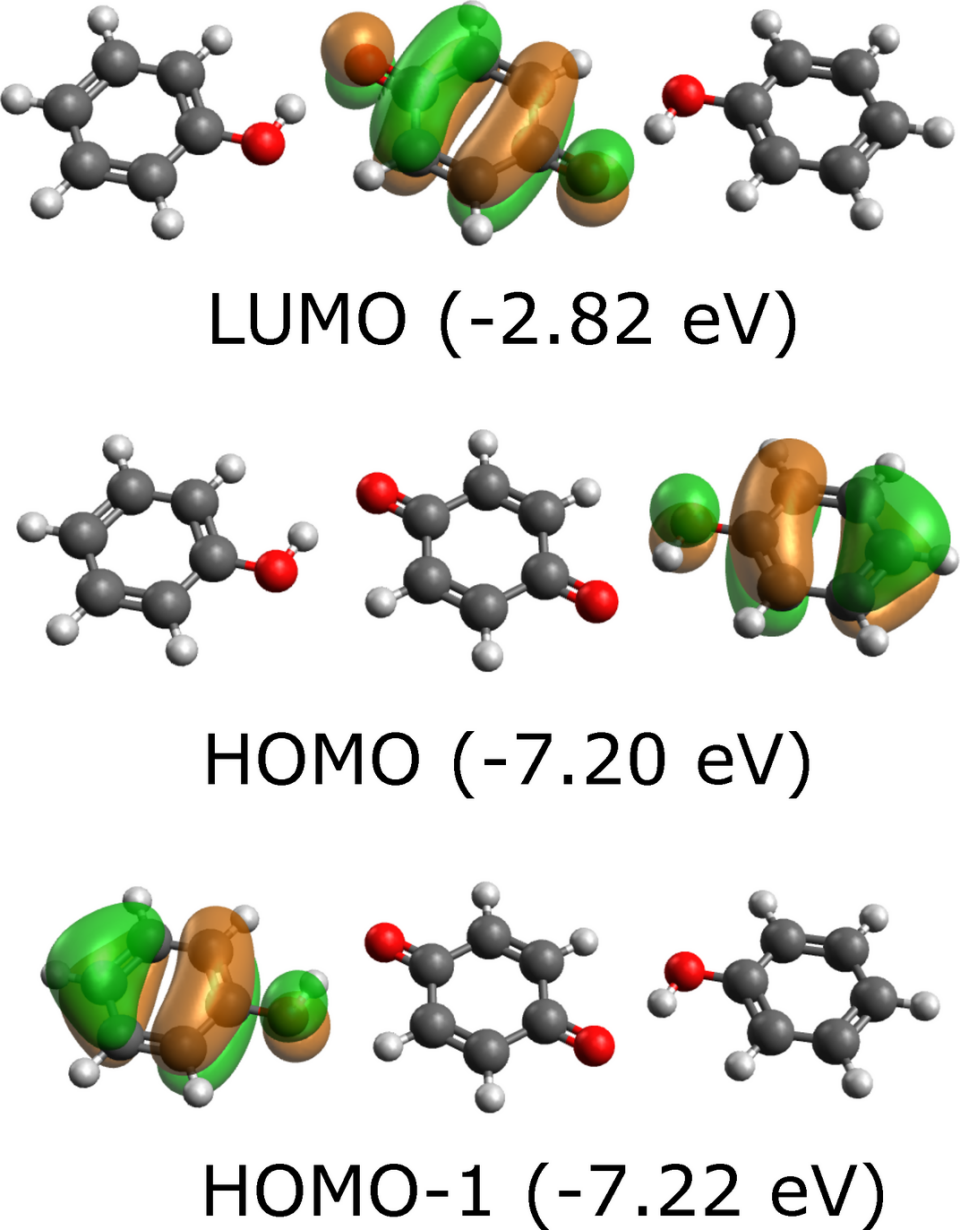
Near-degenerate HOMO and HOMO-1 orbitals and LUMO orbital of pheno­quinone.

Time-dependent density functional theory (TD-DFT) calculations on pheno­quinone reveal a (vibrationally coupled) charge transfer excitation responsible for its color. This type of behavior has been observed in the very similar quinhydrone (Rury *et al.*, 2017 ▸). The first excited state for *p*-benzo­quinone gives rise to a HOMO–LUMO transition at 436 nm, accounting for its yellow color; the HOMO and LUMO of *p*-benzo­quinone are shown in Fig. 4 ▸. Meanwhile, the two lowest excitations for pheno­quinone occur at 530 and 535 nm and correspond to the HOMO-1 to LUMO and HOMO to LUMO transitions, respectively. This accounts for its red color. We note that the HOMO-1 and HOMO of pheno­quinone are quasidegenerate π-anti­bonding orbitals predominantly on each of the phenols, with an energy difference of only 0.016 eV. The HOMO, HOMO-1 and LUMO orbitals of pheno­quinone are shown in Fig. 5 ▸. The HOMO and HOMO-1 orbital are not exactly degenerate because the DFT geometry optimization of pheno­quinone was carried out without any symmetry constraints, resulting in a slightly lower symmetry energy minimum. In the optimized structure, the two O—H bonds differ slightly in length (0.97247 *vs* 0.97264 Å, within the margin of error of the X-ray measurement); this difference is sufficient to lift the degeneracy of the orbital energies in the DFT calculation.

Compared to the intra­molecular HOMO–LUMO transition in *p*-benzo­quinone, the HOMO–LUMO transition in pheno­quinone is an inter­molecular charge-transfer band from much higher energy HOMO levels on the phenols, to the same LUMO predominantly on *p*-benzo­quinone that now has a slightly lower energy due to the cooperative effect of the two hydrogen-bonded phenols. The upshot is that the HOMO-LUMO bandgap is significantly higher in *p*-benzo­quinone (yellow) than in pheno­quinone (red), accounting for the color change that occurs upon formation of the hydrogen-bonded complex.

## 4. Database survey

The structure of the pheno­quinone cocrystal was first determined at room temperature (Sakurai, 1968 ▸) and deposited in the CSD more than 50 years ago as refcode PHENQU (CCDC No. 1232408). Prior to that, Wallwork and Harding had studied this cocrystal (Wallwork & Harding, 1953 ▸; Harding & Wallwork, 1953 ▸), but no crystal structure from their work was deposited in the CSD. Additionally, the quality of the previously obtained structural information was relatively poor because of the primitive nature of diffraction equipment at the time, warranting a re-examination with modern diffraction methods at low temperature.

## 5. Synthesis and crystallization

Both phenol and *p*-benzo­quinone were obtained from Acros Organics. Phenol was used as received, but *p*-benzo­quinone was recrystallized by sublimation in air at 313 K before use. To a nitro­gen-filled test tube sealed with a septum were added 0.109 g of *p*-benzo­quinone (1 mmol) and 0.188 g of phenol (2 mmol). Upon gentle heating with a heat gun, the two solids melted together, forming a clear red liquid. The test tube containing the liquid was placed in a 313 K sand bath to cool, producing a solid mass of red laths and needles of pheno­quinone several mm long within 10 minutes. The crystals thus produced were of X-ray quality. No trace of yellow *p*-benzo­quinone or colorless phenol crystals were observed in the bulk, indicating complete conversion of these starting materials to red pheno­quinone. See the *Supporting Information* for photomicrographs of the single crystal used for structure determination and of a bulk sample of pheno­quinone.

## 6. Density functional theory calculations

The singlet ground-state structures for 1,4-benzo­quinone and the pheno­quinone complex were optimized at the M06-2X/ma-def2-SVP level of theory and basis set (Zhao & Truhlar, 2008 ▸; Zheng *et al.*, 2011 ▸). Dispersion inter­actions were accounted for using Grimme’s D3 dispersion correction with zero damping (Grimme *et al.*, 2011 ▸). Both def2/J and def2-SVP/C auxiliary basis sets were used in the RIJCOSX approximation to speed up the calculation (Neese *et al.*, 2009 ▸). From the optimized geometries, vertical excitation energies were computed using time-dependent density functional theory (TD-DFT) within the Tamm–Dancoff approximation (TDA; Hirata & Head-Gordon, 1999 ▸) at the mPW2PLYP/ma-def2-TZVP level of theory and basis set, employing def2/J and def2-TZVP/C auxiliary basis sets. All electronic structure calculations were performed using the *ORCA 6.0* suite of programs (Neese, 2022 ▸).

## 7. Refinement

Crystal data, data collection and structure refinement details are summarized in Table 1 ▸. The O-bound H atom was freely refined. C-bound H atoms were positioned geometrically (C—H = 0.95 Å) and refined as riding with *U*_iso_(H) = 1.2*U*_eq_(C).

## Supplementary Material

Crystal structure: contains datablock(s) I. DOI: 10.1107/S2056989026005414/ev2026sup1.cif

Structure factors: contains datablock(s) I. DOI: 10.1107/S2056989026005414/ev2026Isup2.hkl

Supporting information file. DOI: 10.1107/S2056989026005414/ev2026Isup3.mol

Photomicrograph of the single crystal used for structure determination; for reference, the loop in the photo is ca. 0.5 mm in diameter. DOI: 10.1107/S2056989026005414/ev2026sup4.jpg

Photomicrograph of a bulk sample of phenoquinone; note the absence of colorless crystals of phenol or yellow crystals of p-benzoquinone. DOI: 10.1107/S2056989026005414/ev2026sup5.png

CCDC reference: 2556203

Additional supporting information:  crystallographic information; 3D view; checkCIF report

## Figures and Tables

**Table 1:** Experimental details

Crystal data	
Chemical formula	C_6_H_4_O_2_·2C_6_H_6_O
*M*_r_	296.31
Crystal system, space group	Monoclinic, *P*2_1_/*c*
Temperature (K)	100
*a*, *b*, *c* (Å)	10.924 (4), 5.905 (2), 11.429 (4)
β (°)	100.203 (7)
*V* (Å^3^)	725.5 (5)
*Z*	2
Radiation type	Mo *K*α
μ (mm^−1^)	0.10
Crystal size (mm)	0.47 × 0.28 × 0.14

Data collection	
Diffractometer	Bruker *APEX*
Absorption correction	Multi-scan (*SADABS*; Krause *et al.*, 2015 ▸)
*T*_min_, *T*_max_	0.678, 0.746
No. of measured, independent and observed [*I* > 2σ(*I*)] reflections	8910, 2218, 1505
*R*_int_	0.053
(sin θ/λ)_max_ (Å^−1^)	0.714

Refinement	
*R*[*F*^2^ > 2σ(*F*^2^)], *wR*(*F*^2^), *S*	0.048, 0.130, 1.04
No. of reflections	2218
No. of parameters	104
H-atom treatment	H atoms treated by a mixture of independent and constrained refinement
Δρ_max_, Δρ_min_ (e Å^−3^)	0.39, −0.31

Computer programs: *APEX2* and *SAINT* V8.40B (Bruker, 2016 ▸), *SHELXT2018/2* (Sheldrick, 2015*a* ▸), *SHELXL2019/3* (Sheldrick, 2015*b* ▸) abnd *OLEX2* 1.5 (Dolomanov *et al.*, 2009 ▸).

## supplementary crystallographic information

### Cyclohexa-2,5-diene-1,4-dione; phenol; phenol Crystal data

**Table:**

C_6_H_4_O_2_·2C_6_H_6_O	*F*(000) = 312
*M**_r_* = 296.31	*D*_x_ = 1.356 Mg m^−^^3^
Monoclinic, *P*2_1_/*c*	Mo *K*α radiation, λ = 0.71073 Å
*a* = 10.924 (4) Å	Cell parameters from 1314 reflections
*b* = 5.905 (2) Å	θ = 3.6–29.9°
*c* = 11.429 (4) Å	µ = 0.10 mm^−^^1^
β = 100.203 (7)°	*T* = 100 K
*V* = 725.5 (5) Å^3^	Needle, clear dark red
*Z* = 2	0.47 × 0.28 × 0.14 mm

### Cyclohexa-2,5-diene-1,4-dione; phenol; phenol Data collection

**Table:**

Bruker APEX diffractometer	2218 independent reflections
Radiation source: sealed tube	1505 reflections with *I* > 2σ(*I*)
Graphite monochromator	*R*_int_ = 0.053
Detector resolution: 8 pixels mm^-1^	θ_max_ = 30.5°, θ_min_ = 1.9°
ω and φ scans	*h* = −15→15
Absorption correction: multi-scan (SADABS; Krause *et al.*, 2015)	*k* = −8→8
*T*_min_ = 0.678, *T*_max_ = 0.746	*l* = −16→14
8910 measured reflections	

### Cyclohexa-2,5-diene-1,4-dione; phenol; phenol Refinement

**Table:**

Refinement on *F*^2^	Primary atom site location: dual
Least-squares matrix: full	Hydrogen site location: mixed
*R*[*F*^2^ > 2σ(*F*^2^)] = 0.048	H atoms treated by a mixture of independent and constrained refinement
*wR*(*F*^2^) = 0.130	*w* = 1/[σ^2^(*F*_o_^2^) + (0.055*P*)^2^ + 0.155*P*] where *P* = (*F*_o_^2^ + 2*F*_c_^2^)/3
*S* = 1.04	(Δ/σ)_max_ < 0.001
2218 reflections	Δρ_max_ = 0.39 e Å^−^^3^
104 parameters	Δρ_min_ = −0.31 e Å^−^^3^
0 restraints	

### Cyclohexa-2,5-diene-1,4-dione; phenol; phenol Special details

**Table:**

Geometry. All esds (except the esd in the dihedral angle between two l.s. planes) are estimated using the full covariance matrix. The cell esds are taken into account individually in the estimation of esds in distances, angles and torsion angles; correlations between esds in cell parameters are only used when they are defined by crystal symmetry. An approximate (isotropic) treatment of cell esds is used for estimating esds involving l.s. planes.

### Cyclohexa-2,5-diene-1,4-dione; phenol; phenol Fractional atomic coordinates and isotropic or equivalent isotropic displacement parameters (Å^2^)

**Table:**

	*x*	*y*	*z*	*U*_iso_*/*U*_eq_	
O1	0.72283 (10)	0.67446 (16)	0.52280 (9)	0.0179 (2)	
O2	0.59468 (9)	0.35765 (15)	0.62685 (8)	0.0171 (2)	
C7	0.55232 (12)	0.1912 (2)	0.56813 (11)	0.0127 (3)	
C1	0.77840 (13)	0.8436 (2)	0.59478 (12)	0.0141 (3)	
C8	0.48755 (12)	0.0104 (2)	0.62234 (11)	0.0136 (3)	
H8	0.481206	0.020546	0.704008	0.016*	
C6	0.77270 (12)	0.8548 (2)	0.71600 (11)	0.0146 (3)	
H6	0.730611	0.740504	0.751912	0.018*	
C9	0.43756 (12)	−0.1677 (2)	0.55862 (11)	0.0133 (3)	
H9	0.395068	−0.280318	0.595305	0.016*	
C2	0.84129 (13)	1.0113 (2)	0.54273 (12)	0.0154 (3)	
H2	0.845732	1.003324	0.460568	0.019*	
C5	0.82913 (13)	1.0347 (2)	0.78352 (12)	0.0165 (3)	
H5	0.825310	1.042669	0.865823	0.020*	
C3	0.89727 (13)	1.1896 (2)	0.61127 (13)	0.0173 (3)	
H3	0.940093	1.303372	0.575684	0.021*	
C4	0.89117 (13)	1.2033 (2)	0.73188 (13)	0.0181 (3)	
H4	0.928956	1.326279	0.778371	0.022*	
H1	0.684 (2)	0.576 (4)	0.5633 (18)	0.055 (6)*	

### Cyclohexa-2,5-diene-1,4-dione; phenol; phenol Atomic displacement parameters (Å^2^)

**Table:**

	*U*^11^	*U*^22^	*U*^33^	*U*^12^	*U*^13^	*U*^23^
O1	0.0218 (6)	0.0144 (4)	0.0177 (5)	−0.0035 (4)	0.0041 (4)	−0.0035 (4)
O2	0.0186 (5)	0.0143 (4)	0.0182 (5)	−0.0015 (4)	0.0029 (4)	−0.0045 (4)
C7	0.0115 (6)	0.0119 (5)	0.0141 (6)	0.0013 (5)	0.0009 (5)	0.0001 (5)
C1	0.0131 (6)	0.0126 (6)	0.0158 (6)	0.0005 (5)	0.0006 (5)	−0.0004 (5)
C8	0.0147 (6)	0.0164 (6)	0.0103 (6)	0.0000 (5)	0.0039 (5)	0.0003 (4)
C6	0.0128 (6)	0.0153 (6)	0.0158 (6)	0.0004 (5)	0.0029 (5)	0.0025 (5)
C9	0.0138 (6)	0.0125 (6)	0.0138 (6)	−0.0003 (5)	0.0033 (5)	0.0023 (5)
C2	0.0146 (7)	0.0180 (6)	0.0139 (6)	0.0013 (5)	0.0031 (5)	0.0016 (5)
C5	0.0140 (7)	0.0211 (6)	0.0139 (6)	0.0023 (5)	0.0013 (5)	0.0000 (5)
C3	0.0133 (7)	0.0164 (6)	0.0223 (7)	−0.0018 (5)	0.0029 (5)	0.0034 (5)
C4	0.0149 (7)	0.0173 (6)	0.0206 (7)	−0.0005 (5)	−0.0006 (5)	−0.0022 (5)

### Cyclohexa-2,5-diene-1,4-dione; phenol; phenol Geometric parameters (Å, º)

**Table:**

O1—C1	1.3657 (15)	C6—C5	1.3910 (18)
O1—H1	0.90 (2)	C9—H9	0.9500
O2—C7	1.2329 (15)	C2—H2	0.9500
C7—C8	1.4764 (18)	C2—C3	1.3880 (19)
C7—C9^i^	1.4787 (18)	C5—H5	0.9500
C1—C6	1.3994 (19)	C5—C4	1.393 (2)
C1—C2	1.3969 (19)	C3—H3	0.9500
C8—H8	0.9500	C3—C4	1.394 (2)
C8—C9	1.3390 (18)	C4—H4	0.9500
C6—H6	0.9500		
			
C1—O1—H1	111.4 (13)	C8—C9—C7^i^	120.83 (11)
O2—C7—C8	120.63 (12)	C8—C9—H9	119.6
O2—C7—C9^i^	121.47 (12)	C1—C2—H2	120.1
C8—C7—C9^i^	117.88 (11)	C3—C2—C1	119.89 (12)
O1—C1—C6	122.55 (12)	C3—C2—H2	120.1
O1—C1—C2	117.49 (12)	C6—C5—H5	119.6
C2—C1—C6	119.95 (12)	C6—C5—C4	120.77 (12)
C7—C8—H8	119.4	C4—C5—H5	119.6
C9—C8—C7	121.28 (12)	C2—C3—H3	119.7
C9—C8—H8	119.4	C2—C3—C4	120.55 (13)
C1—C6—H6	120.2	C4—C3—H3	119.7
C5—C6—C1	119.51 (12)	C5—C4—C3	119.33 (13)
C5—C6—H6	120.2	C5—C4—H4	120.3
C7^i^—C9—H9	119.6	C3—C4—H4	120.3
			
O1—C1—C6—C5	−178.57 (12)	C6—C1—C2—C3	−0.4 (2)
O1—C1—C2—C3	178.69 (12)	C6—C5—C4—C3	−0.5 (2)
O2—C7—C8—C9	177.81 (12)	C9^i^—C7—C8—C9	−1.1 (2)
C7—C8—C9—C7^i^	1.1 (2)	C2—C1—C6—C5	0.51 (19)
C1—C6—C5—C4	0.0 (2)	C2—C3—C4—C5	0.6 (2)
C1—C2—C3—C4	−0.1 (2)		

Symmetry code: (i) −*x*+1, −*y*, −*z*+1.

### Cyclohexa-2,5-diene-1,4-dione; phenol; phenol Hydrogen-bond geometry (Å, º)

**Table:**

*D*—H···*A*	*D*—H	H···*A*	*D*···*A*	*D*—H···*A*
O1—H1···O2	0.90 (2)	1.84 (2)	2.7318 (15)	172.3 (19)

## References

[bb1] Braga, D., Maini, L. & Grepioni, F. (2013). *Chem. Soc. Rev.* **42**, 7638–7648. 10.1039/c3cs60014a 23549606

[bb2] Bruker (2016). *APEX2* and *SAINT*. Bruker AXS Inc., Madison, Wisconsin, USA.

[bb3] Chettri, A., Subba, A., Singh, G. P. & Bag, P. P. (2024). *J. Pharm. Pharmacol.* **76**, 1–12. 10.1093/jpp/rgad097 37934904

[bb4] Dolomanov, O. V., Bourhis, L. J., Gildea, R. J., Howard, J. A. K. & Puschmann, H. (2009). *J. Appl. Cryst.* **42**, 339–341.

[bb5] Duarte, Í., Andrade, R., Pinto, J. F. & Temtem, M. (2016). *Int. J. Pharm.* **506**, 68–78. 10.1016/j.ijpharm.2016.04.010 27073084

[bb6] Fukushima, K. & Sakurada, M. (1976). *J. Phys. Chem.* **80**, 1367–1373.

[bb7] Grimme, S., Ehrlich, S. & Goerigk, L. (2011). *J. Comput. Chem.* **32**, 1456–1465. 10.1002/jcc.21759 21370243

[bb8] Harding, T. T. & Wallwork, S. C. (1953). *Acta Cryst.* **6**, 791–796.

[bb9] Hirata, S. & Head-Gordon, M. (1999). *Chem. Phys. Lett.* **314**, 291–299.

[bb10] Karimi-Jafari, M., Padrela, L., Walker, G. M. & Croker, D. M. (2018). *Cryst. Growth Des.* **18**, 6370–6387.

[bb11] Krause, L., Herbst-Irmer, R., Sheldrick, G. M. & Stalke, D. (2015). *J. Appl. Cryst.* **48**, 3–10. 10.1107/S1600576714022985 PMC4453166 26089746

[bb12] Narala, S., Nyavanandi, D., Srinivasan, P., Mandati, P., Bandari, S. & Repka, M. (2021). *J. Drug. Deliv. Sci. Technol.* **61**, 102209. 10.1016/j.jddst.2020.102209 PMC7946067 33717230

[bb13] Neese, F. (2022). *WIREs Comput. Mol. Sci.* **12**, e1606.

[bb14] Neese, F., Wennmohs, F., Hansen, A. & Becker, U. (2009). *Chem. Phys.* **356**, 98–109.

[bb15] Pratt, D. S. & Gibbs, H. D. (1913). *Philipp. J. Sci., Sect. A* **8**, 51–57.

[bb16] Qi, L., Li, C., Cheng, X., Hao, H. & Xie, C. (2024). *Cryst. Growth Des.* **24**, 6196–6203.

[bb17] Rury, A. S., Sorenson, S. A. & Dawlaty, J. M. (2017). *J. Phys. Chem. Lett.* **8**, 181–187. 10.1021/acs.jpclett.6b02523 27966984

[bb18] Sakurai, T. (1968). *Acta Cryst.* B**24**, 403–412.

[bb19] Sheldrick, G. M. (2015*a*). *Acta Cryst.* A**71**, 3–8.

[bb20] Sheldrick, G. M. (2015*b*). *Acta Cryst.* C**71**, 3–8.

[bb21] Tronov, B. V. & Sokolovich, V. B. (1956). *Izvest. Tomsk. Politekh. Inst.* **83**, 91–97.

[bb22] Vishweshwar, P., McMahon, J. A., Bis, J. A. & Zaworotko, M. J. (2006). *J. Pharm. Sci.* **95**, 499–516. 10.1002/jps.20578 16444755

[bb23] Wallwork, S. C. & Harding, T. T. (1953). *Nature* **171**, 40–41.

[bb24] Yadav, A. V., Shete, A. S., Dabke, A. P., Kulkarni, P. V. & Sakhare, S. S. (2009). *Indian J. Pharm. Sci.* **71**, 359–370. 10.4103/0250-474X.57283 PMC2865806 20502540

[bb25] Zhao, Y. & Truhlar, D. G. (2008). *Theor. Chem. Acc.* **120**, 215–241.

[bb26] Zheng, J., Xu, X. & Truhlar, D. G. (2011). *Theor. Chem. Acc.* **128**, 295–305.

[bb27] Zuev, V. E. (1956). *Trudy Sibir. Fiz.-Tekh. Inst., Tomsk. Univ. im. V. V. Kuibysheva* **35**, 246–248.

