# Supplementary figures and images for: Solvent-free synthesis and low-temperature crystal structure of pheno­quinone (the 1:2 p-benzo­quinone–phenol complex)
Source: Acta Crystallogr E Crystallogr Commun. 2026 Jun 2;82(Pt 7):781–5. doi: 10.1107/S2056989026005414 (PMC13330906; doi:10.1107/S2056989026005414)

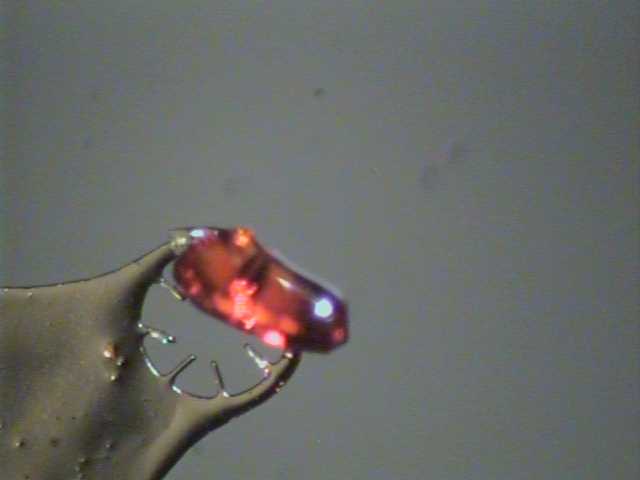

Supplement: Supplementary file 4 [file e-82-00781-sup4.jpg]

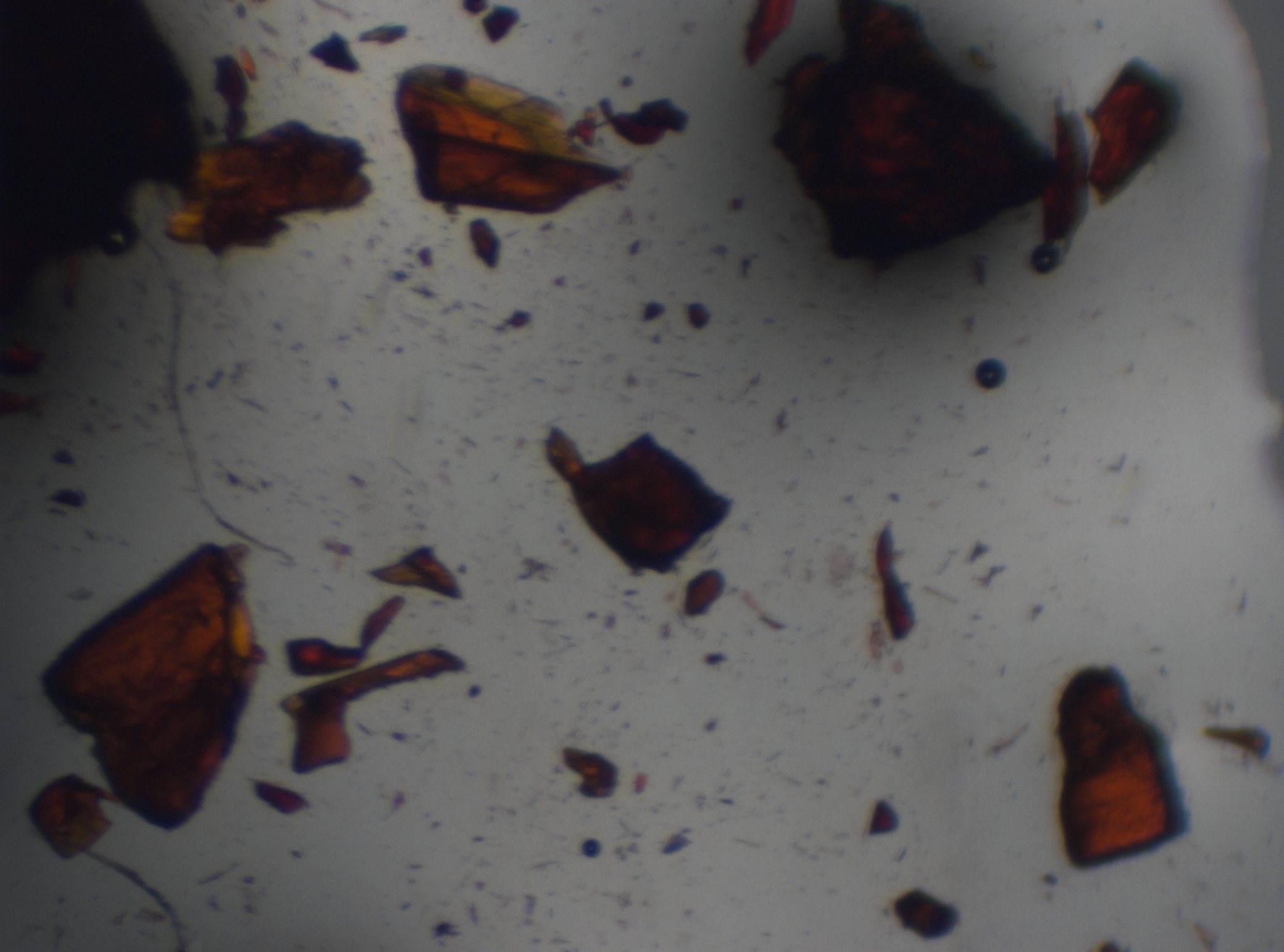

Supplement: Supplementary file 5 [file e-82-00781-sup5.png]
